# Objective Severity Classification of Unilateral Cleft Lip Nasal Deformity Using Nostril Width, Alar Facial Symmetry Ratio, and Columellar Angle to Justify Surgical Outcomes

**DOI:** 10.1007/s00266-025-05082-6

**Published:** 2025-09-04

**Authors:** Pauline Yap, Theddeus Octavianus Hari Prasetyono, Wan Azman Wan Sulaiman, Nurul Syazana Mohamad Shah, Siti Fatimah Noor Mat Johar, Normala Basiron, Shawaltul Akhma Harun Nor Rashid, Ahmad Sukari Halim, Ho Hui Lian

**Affiliations:** 1 Department of Surgery, Faculty of Medicine and Health Sciences, University Putra Malaysia, 43400 Serdang, Selangor Malaysia; 2 Division of Plastic Surgery, Department of Surgery, Cipto Mangunkusumo Hospital/Faculty of Medicine, Universitas Indonesia, Jakarta, Indonesia; 3 ICTEC (Indonesian Clinical Training and Education Center), Cipto Mangunkusumo Hospital/Faculty of Medicine, Universitas Indonesia, Jakarta, Indonesia; 4 Reconstructive Sciences Unit, School of Medical Sciences, Universiti Sains Malaysia, Health Campus, 16150 Kubang Kerian, Kota Bharu, Kelantan Malaysia; 5 Hospital Universiti Sains Malaysia, 16150 Kubang Kerian, Kota Bharu, Kelantan Malaysia; 6 Plastic & Reconstructive Surgery Department, Hospital Kuala Lumpur, 50586 Kuala Lumpur, Malaysia; 7 Department of Surgery, Pusat Perubatan Universiti Kebangsaan Malaysia, Kuala Lumpur, Malaysia; 8 Plastic Surgery Unit, Department of Surgery, Hospital Taiping, Perak, Malaysia; 9 Faculty of Medicine and Health Sciences (FMHS), Plastic, Aesthetic and Reconstructive Surgery Unit, Universiti Malaysia Sabah, 88400 Kota Kinabalu, Sabah Malaysia

**Keywords:** Cleft lip, Nasal deformities, Congenital, Severity classification of unilateral cleft lip and palate, Surgical outcome assessment, Anthropometry

## Abstract

**Introduction:**

Unilateral cleft lip nasal (UCL/N) deformity severity presents complex anatomical challenges, with surgical outcomes influenced by preoperative status. Existing classification systems for UCL/N lack standardization, relying on subjective clinical assessments or isolated anthropometric measures that fails to assess anatomical deformity comprehensively. This limits their utility in objectively stratifying deformity severity and justifying postoperative outcomes. To address this gap, we developed UCL/N Deformity Rating Scale (DRS), an objective classification tool incorporating three validated anatomical indicators: *alar facial symmetry ratio*, *nostril width ratio*, and *columellar angle*. This study aims to (1) validate the scale’s reliability for grading preoperative anatomical severity and (2) establish its clinical applicability by correlating preoperative grades with postoperative aesthetic outcomes, thereby justifying surgical outcomes, not evaluating institutional surgical performance.

**Methods:**

This retrospective cohort study (Level III evidence) analyzed standardized, ethically approved two-dimensional photographs from 50 UCL/N pre- and postoperative patients. All photographs were deidentified, randomized, and independently assessed by three consultant plastic surgeons and three trainees to evaluate interrater reliability using the intraclass correlation coefficient (ICC). The DRS was applied to grade pre- and postoperative severity, and 12 paired cases were analyzed to correlate these grades with postoperative aesthetic outcomes, demonstrating the justification system’s clinical applicability.

**Results:**

Assessments of key anatomical indicators showed consistent agreement across pre- and postoperative evaluations, with no significant differences (p > 0.05). The UCL/N DRS demonstrated good interrater reliability (ICC = 0.64–0.72). In 12 cases, outcomes matched or surpassed predictions: 5 matched, 7 exceeded, and none were substandard. This demonstrates the system’s clinical applicability as a standardized tool to grade primary deformity severity and justify surgical outcomes by linking preoperative severity to postoperative results.

**Conclusions:**

Strong interrater reliability across assessors support the UCL/N DRS as a reliable objective tool for assessing the UCL/N anatomical severity and justifying the post-operative outcome.

**Level of Evidence III:**

This journal requires that authors assign a level of evidence to each article. For a full description of these Evidence-Based Medicine ratings, please refer to the Table of Contents or the online Instructions to Authors www.springer.com/00266.

## Introduction

The severity of primary unilateral cleft lip nose (UCL/N) deformity is a key determinant of postoperative aesthetic outcomes, alongside factors such as surgical expertise, postoperative care, complications, patient load, and healthcare organization [1]. Fisher notes in his exquisite paper on the correlation of objective measures and the severity of the cleft lip deformity, “The eventual look of the lip and nose is governed by a variety of determinants; nevertheless, the key factor is the degree of the primary abnormality” [2].

Studies on surgical outcomes require standardized methods to document primary anatomical abnormalities. Despite numerous attempts to define phenotypic variations and objective indicators for UCL/N severity, no consensus exists on grading or outcome justification [2–6]. Existing tools vary in study design, patient demographics, treatment stages, cleft types, and assessor expertise, leading to inconsistencies and limited reproducibility [5, 7]. Hence, the true impact of primary UCL/N severity on surgical outcomes remains uncertain.

To address this gap, this study introduces and validates the UCL/N Deformity Rating Scale (DRS) as an objective classification system for preoperative severity assessment and surgical outcome evaluation. Unlike prior methods, the DRS offers a structured, reproducible framework, minimizing subjectivity. This study primarily focuses on validating the DRS, with surgical outcome analysis serving to illustrate its clinical applicability rather than assess institutional performance.

## Methods

A scoring system for UCL/N deformity has been established based on three indicators: alar facial symmetry ratio, nostril width ratio, and columellar angle (Table 1). These three indicators are employed because they are readily available and easy to test on a patient, either directly or indirectly via photographs of the patient. Due to the varying distances from which the photographs were taken, ratios were used rather than direct measurements. We aggregate the findings of each measurement to arrive at a total score of between 3 and 9. Primary anatomical severity score is classified into mild, moderate, and severe for each of the variables (Table 1). Preoperative UCL/N anatomical deformity severity is graded into I to IV based on the sum (∑) of UCL/N deformity rating scale (Table 2). The expected postoperative patients’ aesthetic outcome based on the preoperative ∑ UCL/N deformity rating scale is classified into poor, moderate, good, and excellent (Table 3). Postoperative photographs were assessed using the same UCL/N nasal rating scale; the actual postoperative outcome is categorized into poor, moderate, good and excellent result (Table 4). Subsequently, the actual outcome is compared with the expected outcome and the outcome justification system is proposed. The proposed outcome justification system (Table 5) is utilized to determine whether the outcome obtained is adequate or substandard.

**Table 1:** UCL/N Deformity Rating Scale

Variable	Class	Score
Alar facial symmetry ratio	Mild (0.01-0.05) Moderate (>0.05-0.10) Severe (>0.10)	3 2 1
Nostril width ratio	Mild (0.90-1.10) Moderate (0.60-<0.90; >1.10-1.40) Severe (<0.60; >1.40)	3 2 1
Columellar angle (degree)	Mild (0-15) Moderate (16-30) Severe (>30)	3 2 1

Total score ranges from 3 to 9

**Table 2:** UCL/N deformity severity grade based on ∑ Deformity Rating Scale

∑ UCL/N deformity rating scale	UCL/N deformity severity grade
9	I
7–8	II
4–6	III
3	IV

**Table 3:** Expected UCL/N postoperative outcome rating grade based on preoperative ∑ UCL/N Deformity Rating Scale

Preoperative ∑ UCL/N Deformity Rating Scale	Expected UCL/N postoperative outcome grade
3	Poor
4–6	Moderate
7–8	Good
9	Excellent

**Table 4:** Actual UCL/N postoperative outcome rating grade based on postoperative ∑ UCL/N Deformity Rating Scale

Postoperative ∑ UCL/N Deformity Rating Scale	Actual UCL/N postoperative outcome grade
3	Poor
4–6	Moderate
7–8	Good
9	Excellent

**Table 5:** Justification of UCL/N postoperative outcome rate based on preoperative **∑** UCL/N Deformity Rating Scale

Preoperative ∑ UCL/N Deformity Rating Scale	Justification of UCL/N postoperative outcome grade
3–5	Moderate to Poor
6	Moderate
7–9	Good to Excellent

Preoperative scale 7-9 is not justified to get moderate outcome and no acceptance for poor outcome

This study aims to verify the UCL/N Deformity Rating Scale as a credible tool in providing an objective assessment to classify the primary UCL/N anatomical deformity severity and evaluate surgical outcome. Additional section is provided to demonstrate the clinical application of the outcome justification system developed.

## Statistical Analysis

Statistical analyses were performed using SPSS version 24. Interrater reliability was measured using intraclass correlation coefficients (ICCs) [8]. A sample size of 50 was selected to ensure robust reliability analysis, adhering to methodological guidelines for ICC studies [9].

The subject sample population size of 50 sets was determined based on statistical considerations to ensure adequate power for assessing interrater reliability using the ICC. According to Bujang (2017), with six assessors, a minimum of six subjects is required to achieve a power of 90% for an ICC value of 0.6[9]. To enhance the robustness of our study and improve the precision of the ICC estimates, we opted for a larger sample size. By selecting 50 sets, we accounted for a potential 10% data loss due to unusable images and increased the generalizability of our findings.

Out of 50 sample populations, there were 12 that had paired pre- and postoperative results. The mean value of the 3 indicators measured by the 6 assessors was obtained. The expected and actual postoperative outcome results were compared. Justification system was proposed. How the DRS can be utilized clinically is demonstrated.

### Subjects (Sample Population)

All subjects were recruited from patients admitted for primary cheiloplasty and follow-up between January 2014 and December 2021 at 3 centers, i.e., Pusat Pengajian Sains Perubatan, Health Campus, Universiti Sains Malaysia (USM), Hospital Raja Perempuan Zainab II, Kelantan, and Hospital Kuala Lumpur, Malaysia. Parents or guardians consented to using their medical records and photographs. This study was given ethical clearance by the Jawatankuasa Etika Penyelidikan Manusia USM (JEPeM), with study protocol code USM/JEPeM/20070388.

Photographers captured standardized submental (basal) and anteroposterior (frontal) presurgical and postoperative photographs with a Huawei digital phone camera (Huawei Technologies Co., Ltd., Shenzhen, China) and a Sony digital camera (Tokyo Telecommunications Engineering Corporation, Tokyo, Japan). The patients’ subjects were randomly selected based on the inclusion and exclusion criteria (Table 6).

**Table 6:** Sample populations inclusion and exclusion criteria

Inclusion Criteria	Exclusion Criteria
Male and female	Craniofacial congenital anomalies.
Aged between 2 months and 20 years	Congenital syndromes.
Unilateral cleft lip, with or without accompanying alveolar or palate cleft.	Bilateral cleft lip and palate.

The inclusion of patients aged 2 months to 20 years ensures the study captures outcomes across the typical window for primary cleft lip repair and subsequent follow-up, encompassing infants undergoing primary cheiloplasty and young adults representing long-term results. Syndromic cases were excluded to maintain a homogenous study population, as their cleft deformities are often associated with additional anomalies that could confound the analysis. Similarly, bilateral cleft lip and palate cases were excluded due to their distinct anatomical complexities, which differ significantly from unilateral cases. These differences require separate assessment criteria and methodologies, potentially introducing variability and bias in the analysis. Excluding these cases ensures consistency and comparability in evaluating the UCL/N deformities.

None of the included subjects underwent pre-orthodontic interventions, and the surgical repairs were performed by multiple surgeons. Although the surgical technique was not explicitly standardized as a controlled factor, all surgeons consistently practiced the modified Millard rotation advancement technique. This ensured a level of uniformity in the surgical method. We recognize the potential impact of pre-orthodontic interventions on surgical outcomes; however, this factor was excluded to maintain consistency. Additionally, pre-orthodontic interventions are not commonly practiced in our center for UCL/N patients, making it less relevant to the scope of this study.

**Fig. 1:**
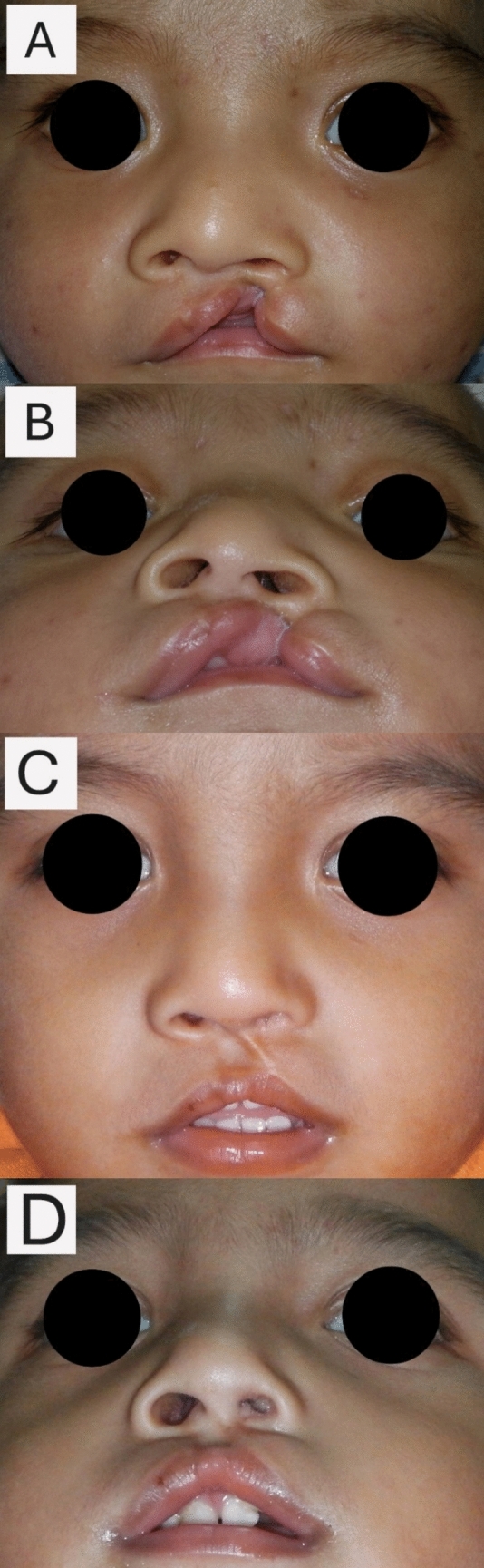
Preoperative **A** and **B** and postoperative **C** and **D** photograph. **A&C** indicates the standard anteroposterior, and **B&D** indicates submental views’ photographs. The photographs were cropped accordingly following the borders: superior margin above the eyebrows and inferior margin at lower border of lower lip. These are photographs taken from the same patient. However, there are slight variations in exposure, as the photographs were taken while the children were awake and in different rooms

From this group, 50 sets of pre- and postoperative photographs were randomly selected and deidentified. Preoperative photographs were taken a day before or immediately prior to the surgery. Postoperative photographs were captured at least 6 months after the surgery. Standard anteroposterior and submental viewed photographs were then cropped accordingly following the borders: superior margin above the eyebrows and inferior margin at oral commissures or the junction between closed lips (Fig. 1). All photographs were color printed on standard A4 paper.

### Assessors

Two groups of assessors were recruited voluntarily by giving consent. The first group comprised of three skilled board-certified plastic surgeons, who have substantial experience with cleft lip and palate correction. The second group consisted of three plastic surgery residents in their third semester or higher, who assisted on at least five cleft lip repair surgeries. The inclusion criteria ensure assessors have sufficient clinical experience and knowledge of UCL/N anatomical deformities for accurate evaluations. Residents in advanced training stages were included to test the system's usability by future practitioners, while plastic surgeons provided expert validation.

### Measurement Techniques for the Three Indicators

**Fig. 2:**
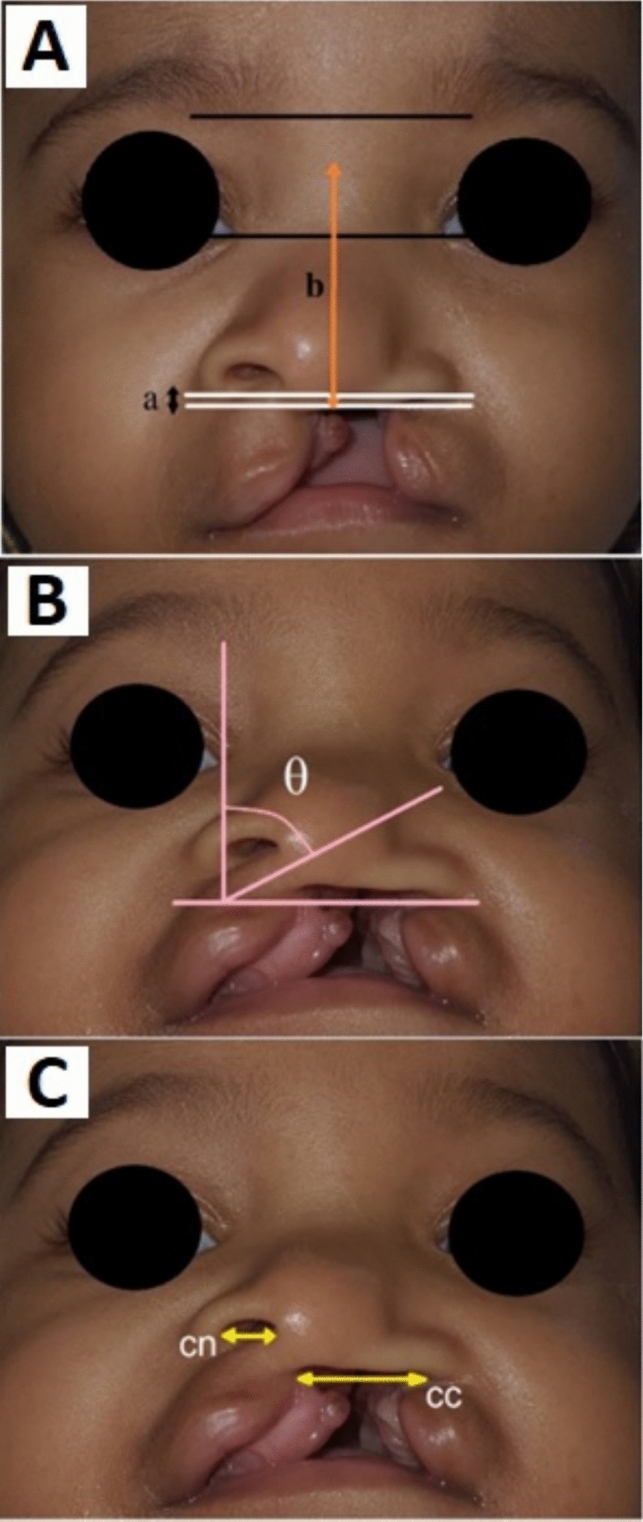
Methods of measurement: To measure alar facial symmetry, the gap between alar facial groove ***a*** was divided by the nasal length ***b*** to generate a ratio (**A**). The vertical gap of alar facial grooves ***a*** is the vertical distance between the shown white lines. Nasal length ***b*** is the length of vertical line connecting the horizontal line which crosses the lower alar base to the nasion shown as orange line in the frontal view (**A**). Columella angle (θ) is measured on the submental view shown in pink lines. The angled (oblique) line is drawn following the axis of the columella. A vertical line is created to meet with the angled line to measure the columellar angle shown in submental view (**B**). Nostril width is the distance between the most medial and lateral borders of the nostril measured on submental view (**C**) (yellow lines). ***a*** gap between alar facial groove; *b* nasal length; *cn* normal nostril’s width; *cc* cleft nostril’s width

Assessors were given sets of standardized photographs, textual and video instructions on indicators measurements, and operational definitions. Participants measured the pre- and postoperative vertical gap of alar facial grooves, nasal length, right and left nostril width, and columellar angle on each set of photographs with reference to Prasetyono et al[10] as indicated in Figure 2.

Two horizontal lines were drawn at the lowest point of the bilateral alar facial grooves on the frontal view photographs of the patients to establish the alar facial symmetry. The distance between those lines was then measured and compared to the nasal length. The alar facial groove marks the juncture between the ala and the cheek, while nasal length represents the measurement of a vertical line connecting the horizontal line that intersects the lower alar base with the midpoint situated between the two horizontal lines crossing the medial canthuses and the lower medial border of the eyebrows [10] (Fig. 2A).

The columellar angle is measured by drawing a vertical line perpendicular to the horizontal line intersecting the alar facial groove to determine submental nasal symmetry. Then, the angle made by the columella from the vertical line is measured [8] (Fig. 2B). Nostril width is the horizontal distance between the innermost and outermost edges of the nostril on submental perspective [8] (Fig. 2C). The nostril widths of the cleft and non-cleft sides were measured and compared to establish the nostril width symmetry.

**Fig. 3:**
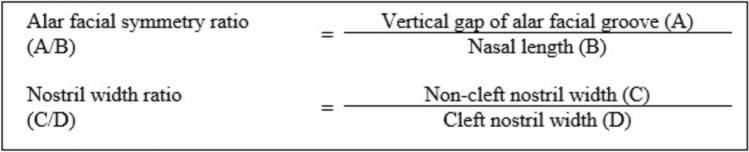
Alar facial symmetry and nostril width ratio calculations formula

**Fig. 4:**
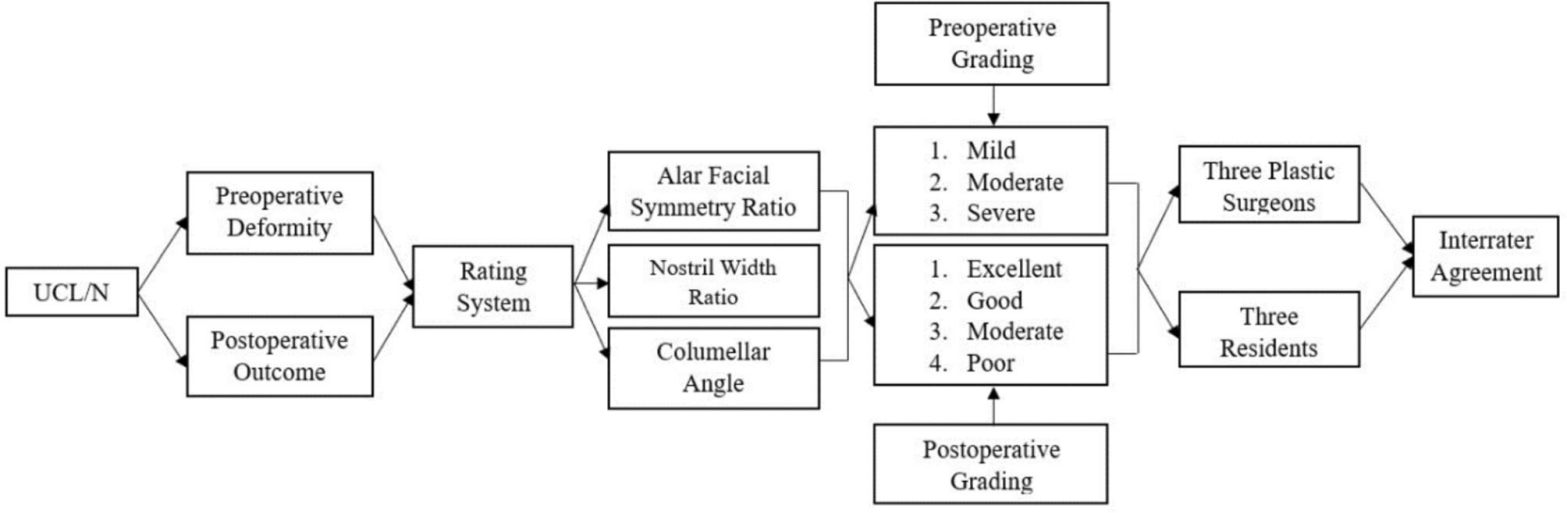
Flowchart illustrating the UCL/N Deformity Rating Scale (DRS) assessment process. It outlines the photographic evaluation of preoperative deformities and postoperative outcomes using three anatomical parameters (alar facial symmetry ratio, nostril width ratio, columellar angle). It also shows how severity and outcome are graded separately, and describes the assessment of interrater reliability involving three plastic surgeons and three residents

The alar facial symmetry ratio (A/B) and nostril width ratio (C/D) ratios were calculated based on the following formula (Fig. 3):

To enhance clarity, a flowchart (Fig. 4) is provided to summarize the structure of the UCL/N rating process, including the anatomical parameters evaluated, grading categories, and interrater reliability assessment.

## Results

### Demographic Data

The patients recruited were exclusively Asian, primarily of Malay ethnicity, with one patient of Indian and one of Chinese descent. Out of the preoperative patients, 44 (88%) fall within the age range of 1 to 6 months, while 3 (6%) are between 6 months and 1 year, and another 3 (6%) are older than 1 year. Out of the total postoperative patient population, 5 (10%) were aged between 6 months and 1 year, 10 (20%) were aged between 1 and 2 years, 18 (36%) were aged between 2 and 5 years, and 17 (34%) were aged above 5 years. The average postoperative follow-up duration is 4 years and 10 months.

### Severity and Cleft Type Analysis

Out of the 50 preoperative patients, we had 31 patients with complete cleft lip and palate (CCLP), 10 had incomplete cleft lip (ICL), and 9 had incomplete cleft lip and alveolus (ICLA), based on our data consolidated in Table 7 and Appendix 2. The high frequency of Grade III cases in CCLP 26(52%) out of 31 cases, along with the presence of 5 cases of Grade IV deformities, suggests that this type of cleft is more likely to result in severe deformities. This observation indicates a potential relationship between the type of cleft and the degree of deformity.

**Table 7:** Distribution of preoperative UCLN severity grades across different cleft types

Grade	Type of Cleft		
	CCLP (Pax/%)	ICL (Pax/%)	ICLA (Pax/%)
I	0	0	0
II	0	6 (12)	1 (2)
III	26 (52)	4 (8)	7 (14)
IV	5 (10)	0	1 (2)

*CCLP* Complete cleft lip and palate; *ICL* Incomplete cleft lip; *ICLA* Incomplete cleft lip with alveolus

ICL cases received grade II of 6 out of 10 cases. This prominence indicates that this type of cleft typically leads to moderate deformities. The absence of Grade IV cases and a smaller number of Grade III cases in ICL suggest that it generally results in less severe deformities compared to CCLP. ICLA shows a more diverse distribution of severity grades, but with a tendency toward Grade III (Severe) severity (7 out of 9 cases). This suggests that ICLA can lead to a range of deformity severities, often resulting in moderate to severe deformities. However, we acknowledge that this conclusion is drawn from observed patterns in the data rather than formal statistical testing. Future research by applying appropriate statistical methods to further validate this observation is needed (Tables 8 and 9).

**Table 8:** Preoperative alar facial symmetry ratio, nostril width ratios and columellar angle measured by the assessor groups

Variables	Surgeon group Mean (SD)	Resident group Mean (SD)	Mean difference (95% CI)	p
Ratio A/B	2.34(0.63)	2.28(0.64)	0.06( −0.19,0.31)	0.637
Ratio C/D	1.18(0.39)	1.36(0.56)	−0.18( −0.37,0.01)	0.066
Columellar angle	1.44(0.64)	1.50(0.71)	−0.06( −0.33,0.21)	0.658

Ratio A/B = alar facial symmetry ratio; Ratio C/D = nostril width ratio

**Table 9:** Postoperative alar facial symmetry ratio, nostril width ratios, and columellar angle measured by the assessor groups

Variables	Surgeon group Mean (SD)	Resident group Mean (SD)	Mean difference (95% CI)	p
Ratio A/B	2.92(0.27)	2.90(0.30)	0.02(-0.09,0.13)	0.730
Ratio C/D	2.26(0.56)	2.20(0.49)	0.06(-0.15,0.27)	0.573
Columellar angle	2.76(0.43)	2.66(0.48)	0.10(-0.08,0.28)	0.275

Ratio A/B = alar facial symmetry ratio; Ratio C/D = nostril width ratio

### Inter-rater Reliability

Our study demonstrates that the DRS reliably measures cleft nasal deformities for both experienced surgeons and trainees. Surgeons and residents produced nearly identical scores across all measurements (Tables 8–9) (refer to Appendix 1), demonstrating consistency among users with relevant cleft training. Statistical tests (Table 10) confirmed strong agreement between assessors, with columellar angle measurements being the most consistent (*ICC* = 0.72), followed by nostril width ratio (*ICC* = 0.69) and alar symmetry (*ICC* = 0.64). These results meet established standards for good agreement [8].

**Table 10:** Intraclass correlation (ICC) for each variables measured

Variables	ICC (95% CI)	p
Ratio A/B	0.64(0.36,0.79)	<0.001
Ratio C/D	0.69(0.44,0.83)	<0.001
Columellar angle	0.72(0.50,0.84)	<0.001

Ratio A/B = alar facial symmetry ratio; Ratio C/D = nostril width ratio

To further clarify interpretation, we refer to Cichetti (1994) [8], who classified ICC values as follows: (Table 11). Using this scale, ICC values produced by our 6 assessors fall within the “Good” agreement category.

**Table 11:** Interpretation scale for intraclass correlation coefficients (ICC) as defined by Cicchetti (1994), commonly used to evaluate interrater reliability. ICC values below 0.40 indicate poor agreement, while values of 0.60 or higher represent good to excellent consistency across raters. The ICC results in this study fall within the “good” range, demonstrating consistent scoring among all six assessors

ICC Value	Strength of Agreement
< 0.40	Poor
0.40–0.59	Fair
0.60–0.74	Good
0.75–1.00	Excellent

### Paired Sample Analysis

#### Demographic Data

Out of a sample population of 50, 12 were paired for comparison of the actual aesthetic outcome to the expected outcome based on the preoperative UCL/N DRS. The paired samples consist exclusively of individuals of Malay ethnicity, evenly divided between female and male. Preoperative photographs were taken when the children were between 1 and 6 months old. Postoperative photographs were captured at various stages: 6 to 12 months old (2 samples), 1 to 2 years old (2 samples), 2 to 5 years old (4 samples), and over 5 years old (4 samples). The preoperative samples average age is 3.6 months, postoperative samples average age is 4 years 1 month, and the average follow-up time is 3 years 11 months (Table 12).

**Table 12:** Rating result of total 6 assessors for each preoperative UCL/N deformity and postoperative outcome on paired photographs of sample population

Preoperative (Median)						Postoperative (Median)			
Sample Population	Ratio A/B (Score)	Ratio C/D (Score)	Columellar Angle (Score)	Preoperative UCL/N deformity rating scale/ severity grade		Ratio A/B (Score)	Ratio C/D (Score)	Columellar Angle (Score)	Actual postoperative UCL/N deformity rating scale (Total score)
				Total score	Grade				
1	0.09(2)	0.41(1)	37.5(1)	4	III	0.04(3)	0.72(2)	9.0(3)	8
2	0.06(2)	0.49(1)	39(1)	4	III	0.02(3)	0.91(3)	16.0(2)	8
3	0.04(3)	0.65(2)	21.5(2)	7	II	0.02(3)	1.18(2)	13.0(3)	8
4	0.04(3)	0.54(1)	26.0(2)	6	III	0.01(3)	0.90(3)	2.0(3)	9
5	0.01(3)	0.63(2)	12.0(3)	8	II	0.03(3)	0.83(2)	0(3)	8
6	0.03(3)	0.71(2)	16.25(2)	7	II	0.01(3)	0.84(2)	4.5(3)	8
7	0.07(2)	0.43(1)	38,83(1)		III	0.02(3)	0.57(1)	23.5(2)	6
8	0.05(3)	0.41(1)	33.5(1)	5	III	0.02(3)	0.97(3)	11.75(3)	9
9	0.14(1)	0.36(1)	50(1)	3	IV	0.02(3)	0.91(3)	21.5(2)	8
10	0.03(3)	0.79(2)	11.75(3)	8	II	0.05(3)	1.38(2)	9.00(3)	8
11	0.02(3)	0.52(1)	52(1)	5	III	0.02(3)	0.72(2)	22(2)	7
12	0.04(3)	0.41(1)	44.5(1)	5	III	0.01(3)	0.94(3)	14.5(3)	9

#### Demonstration of Outcome Justification Analysis

This section demonstrates how the UCL/N DRS can be applied to assess surgical outcomes based on preoperative severity classification. It is not intended as an audit of institutional surgical performance but rather as a demonstration of the justification system’s clinical utility in outcome assessment. Among the 12 paired sample populations, 5 had justified actual outcomes, 7 had actual outcomes that exceeded the justified outcome suggesting exceptional aesthetic results, and none had an unjustified outcome as detailed in Tables 12 and 13. This information indicates that the justification system’s classification effectively accounts for variability in surgical result. It has successfully detected exceptional good result from merely acceptable outcome. The consistency of favorable postoperative outcomes within the expected framework supports the system's reliability in objectively categorizing actual surgical outcomes. In cases where the total pre- and postoperative scores remain the same (e.g., Sample 5 and Sample 10) in Table 13), a “Justified” classification indicates that the surgical outcome aligns with what was expected based on the preoperative severity. This does not imply that the surgery had no impact but rather that the outcome achieved meets the realistic expectations set by the severity of the primary deformity.

**Table 13:** Justification of postoperative aesthetic outcome based on preoperative nasal deformity rating scale on each sample population

Sample Population	Preoperative UCL/N Deformity Rating Scale		Expected postoperative outcome	Postoperative UCL/N Deformity Rating Scale (Total score)	Actual postoperative outcome grade	Justification
	Total score	Grade				
1	4	III	Moderate	8	Good	Better than justified
2	4	III	Moderate	8	Good	Better than justified
3	7	II	Good	8	Good	Justified
4	6	III	Moderate	9	Excellent	Better than justified
5	8	II	Good	8	Good	Justified
6	7	II	Good	8	Good	Justified
7	4	III	Moderate	6	Moderate	Justified
8	5	III	Moderate	9	Excellent	Better than justified
9	3	IV	Poor	8	Good	Better than justified
10	8	II	Good	8	Good	Justified
11	5	III	Moderate	7	Good	Better than justified
12	5	III	Moderate	9	Excellent	Better than justified

Unlike direct numerical comparison, the surgical outcome classification (Tables 3, 4) is derived from the primary severity grading. Based on this grading, severity-adjusted categorizations of expected and actual outcomes are developed using the UCL/N DRS, divided into severity-adjusted descriptive categories (poor, moderate, good, excellent) (Tables 3, 4). This approach replaces numerical comparisons with a qualitative framework.

To prevent over- or underestimation, the outcomes justification classification (Table 5) is derived from readjustment of the original outcome classification (Tables 3, 4): Preoperative scores 3–5 (severe deformities) are justified to moderate–poor outcomes, while scores 7–9 (milder cases) are justified to good–excellent outcomes (Table 5). This ensures realistic expectations, severe cases align with moderate-poor results, whereas milder cases cannot justify moderate-poor outcomes. The justification table (Table 5) directly compares these categorized expectations and actual results to objectively derive clinically meaningful outcome justification.

A common misconception is that unchanged pre- and post-op scores indicate no improvement. The key is to understand that the system does not measure success based on absolute numerical change. Instead, it determines whether the final result is justified relative to the preoperative severity grade, as defined by the severity-adjusted descriptive categories. Severe cases may show minimal numerical change but still achieve an outcome that aligns with realistic expectations, making the result justified. Likewise, less severe cases may exhibit less numerical change, but this does not imply a lack of improvement.

If the postoperative outcome falls outside the expected category, it can indicate one of two possibilities: Either the outcome is better than expected, or it is an unjustified outcome, meaning the result is substandard for that severity level (refer to Table 5). The Justification system ensures that each case is evaluated within its severity-adjusted classification rather than being judged purely by numerical changes. This approach allows for an objective, structured, and reproducible assessment of surgical success, eliminating subjective biases and providing a standardized method for evaluating surgical outcomes.

To enhance clarity, a summary of the preoperative severity classification and outcome justification system is provided (Table 14). This framework links preoperative anatomical severity to expected surgical outcomes, enabling objective assessment of whether surgical results are justified. It sets realistic expectations by adjusting anticipated results according to the severity of the deformity.

**Table 14:** Summary of severity classification and justification framework. This table summarizes how preoperative UCL/N deformity severity scores are classified into Grades I–IV and linked to expected surgical outcomes. The “Justified Outcome Range” defines the acceptable postoperative result for each grade. For example, a patient with a preoperative score of 9 (Grade I) is expected to achieve an excellent result, and outcomes rated as “Good” or “Excellent” would be considered justified. Preoperative score of 9 is not justified to receive a moderate to poor outcome

Preoperative Total Score	Severity Grade	Expected Postoperative Outcome	Justified Outcome Range
9	I	Excellent	Good—Excellent
7–8	II	Good	Good—Excellent
6	III	Moderate	Moderate
4–5	III	Moderate	Moderate—Poor
3	IV	Poor	Moderate—Poor

Preoperative scale 7-9 is not justified to get moderate outcome and no acceptance for poor outcome

**Fig. 5:**
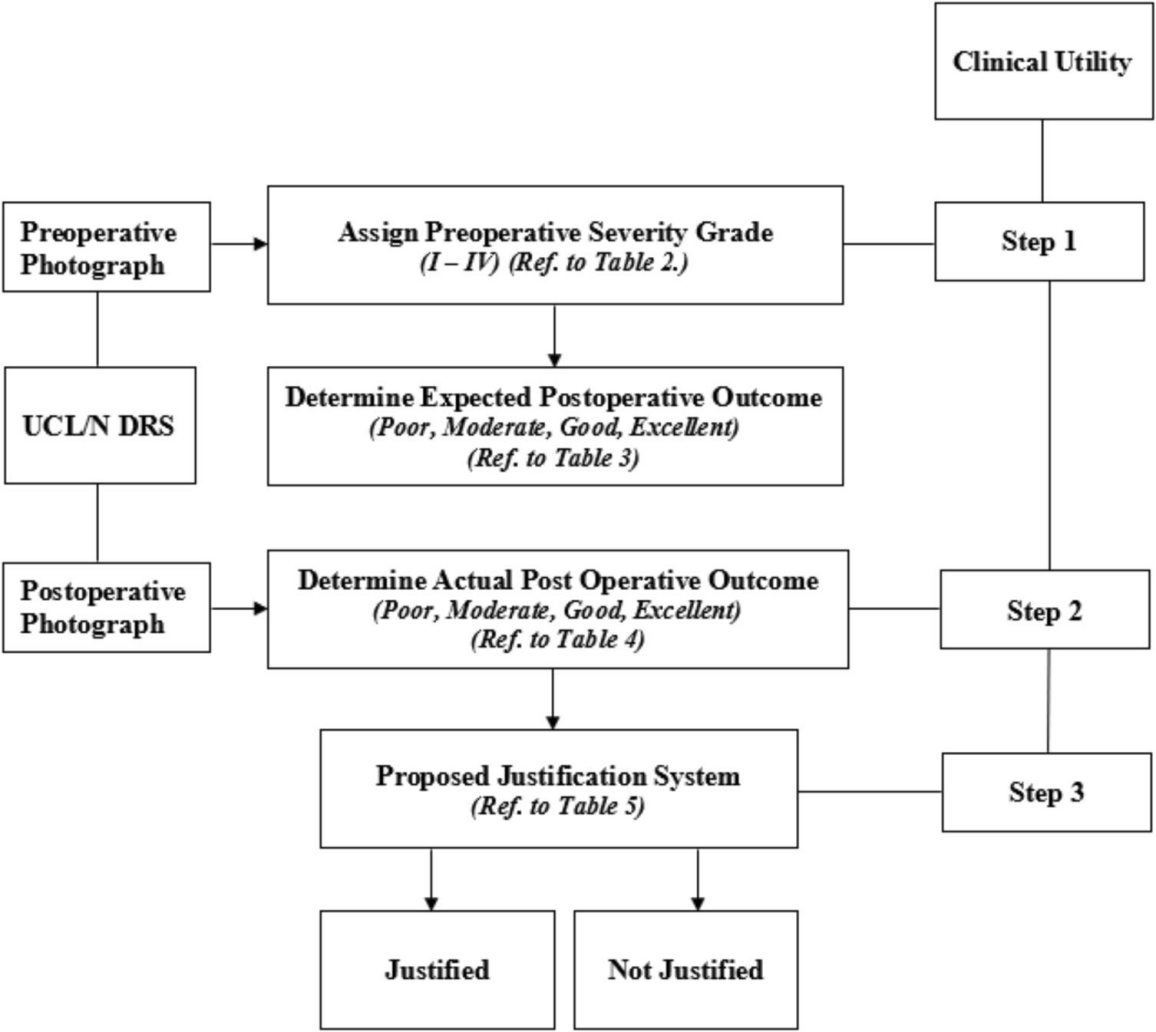
Flowchart illustrating the workflow of the UCL/N Deformity Rating Scale (DRS) application for outcome justification. The clinical utility of this system is demonstrated in three distinct steps: Step 1 involves assigning a severity grade (I–IV) based on preoperative photographs; Step 2 involves assessing and grading the actual postoperative outcomes; Step 3 involves applying the justification system to classify outcomes as either “Justified” or “Not Justified”

A visual flowchart (Fig. 5) illustrates the workflow of the UCL/N DRS clinical application for outcome justification. Step 1 involves assigning a severity grade (I–IV) based on preoperative photographs; Step 2 involves assessing and grading the actual postoperative outcomes; Step 3 involves applying the justification system to classify outcomes as either "Justified" or "Not Justified."

## Discussion

Postoperative aesthetic outcomes in cleft lip repair depend critically on preoperative anatomical severity, particularly the degree of nasal and lip deformity. While surgical expertise and technique influence results, the primary deformity’s severity remains a pivotal prognostic factor. Standardized preoperative assessment is essential to objectively correlate surgical outcomes with primary anatomical severity, enabling comparisons across surgeons, institutions, and techniques.

Fisher et al. demonstrated that expert subjective assessments correlate with objective anthropometric measurements, validating the nostril width ratio as reliable severity indicators[2, 11]. Subsequent studies by Mercan, Yao, He, and colleagues identified additional markers such as columellar angle, nasal alae symmetry, cleft width ratio, and lateral lip height that quantify anatomical distortion [2, 12–16]. Existing tools, such as Yao’s cleft width ratio-based scale [13], lack comprehensiveness in addressing other abnormal UCL/N deformities caused by dynamic anatomical changes arising from aberrant nasal, paranasal muscle attachment, and orbicularis oris discontinuity. These muscle-driven distortions create asymmetric tissue displacement, which is pivotal to UCL/N severity assessment.

The UCL/N Deformity Rating Scale addresses this gap by integrating validated indicators to assess three-dimensional nasal anatomy. This scale accounts for musculofacial dynamics critical to cleft-related asymmetry, providing a holistic framework to grade severity and guide outcome evaluation.

### Novelty of the UCL/N Deformity Rating Scale

#### Rationale for Indicator Selection

The DRS was designed to address the multifaceted anatomical challenges of UCL/N deformity by integrating objective, reproducible measurements aligned with core principles of facial aesthetics: symmetry, proportionality, and anatomical fidelity. Three key indicators: nostril width ratio, columellar angulation, and alar facial symmetry ratio were selected based on their ability to (1) quantify deviations across primary nasal subunits including nostrils, columella, and alar bases, (2) reflect the most impactful anatomical distortions in cleft-related asymmetry, and (3) serve as validated predictors of surgical outcomes while balancing granularity with clinical feasibility[2, 12–17]. Below, we elaborate on their rationale.

#### Nostril Width Ratio

Nostril asymmetry is a hallmark of UCL/N deformity, driven by tissue displacement from abnormal muscles attachments, discontinuity and soft tissue, skeletal hypoplasia [18–20]. The nostril width ratio (cleft/non-cleft width or *vice versa*) directly measures this asymmetry, with an ideal value of 1. Deviations >40% (i.e., ratios >1.4 or <0.6) signify severe asymmetry, while even subtle deviations (±0.1) disrupt facial harmony. This range of ratio scale is based on Fujimoto–Imai method [9, 21]. This ratio is a validated marker of surgical success, guiding interventions to restore symmetry, a critical determinant of patient satisfaction [2, 10, 21–24].

#### Columellar Angle

Columellar angle in the basal view reflects septal alignment and soft tissue integrity. It is a widely recognized indicator to assess primary deformity [2, 16, 22–24]. However, to date there is lack of established explicit grading thresholds for the measurements. A 0° of columellar deviation angle at midline alignment represents the anatomical ideal. We categoried deviations >30° as indicative of severe septal displacement or alar malposition. The DRS classifies severity into 15° increments (mild: <15°, moderate: 15–30°, severe: >30°). This enables surgeons to tailor techniques to address distortions such as abnormal muscle insertions, septal deviation, or alar cartilage malposition.

#### Alar Facial Symmetry Ratio

The alar facial symmetry ratio quantifies the relationship between alar base displacement and nasal length, contextualizing midface disproportionality. In UCL/N, abnormal orbicularis oris muscle insertions and skeletal hypoplasia displace the alar facial groove posteriorly and inferiorly, widening the alar base and disrupting midface harmony. By normalizing the vertical gap between the alar groove (**a**) against nasal length (**b**), this ratio (A/B) provides an objective measure of severity independent of nasal size. Previous study by Prasetyono et al first applied this indicator to determine the outcome of cleft repair [10]. A ratio approaching 0 indicates optimal alignment, while larger values reflect more displacement. This proportional approach ensures clinical relevance across diverse populations, accounting for ethnic and individual variations in nasal morphology.

#### Integration of Validated Parameters

Existing studies have assessed isolated nasal parameters as independent markers of cleft severity [2, 13–17, 24]. The UCL/N Deformity Rating Scale is the first to integrate validated indicators—including nostril width ratio, columellar angulation, and alar facial symmetry ratio—into a single grading system, enabling simultaneous evaluation of nasal subunits. It captures interplay between multiple anatomical distortions: the nostril width ratio quantifies transverse nasal asymmetry [18–20], the columellar angle reflects axial septal deviation [25–28], and the alar facial ratio contextualizes vertical alar displacement relative to nasal length. These integrated parameters enable comprehensive evaluation of cleft nasal deformity by accounting for septal deviation, soft tissue deficiency, skeletal hypoplasia, and secondary changes in nasal cartilage morphology caused by aberrant muscle traction in the absence of nasal floor and orbicularis oris continuity. This multidimensional synthesis of anatomical parameters enables surgical assessment targets root causes of asymmetry rather than isolated distortions, even when measurements are derived from 2D photographs. Improvements in these anatomical parameters are proven to correlate with functional outcomes such as balanced airflow and patient satisfaction [18–20, 29, 30], further validating their role as proxies for surgical success.

#### UCL/N Deformity Rating Scale’s Limitations

The DRS composed of mainly nasal deformity assessment; it excludes lip-specific parameters (e.g., vermilion height, Cupid’s bow symmetry) and other nasal indicators (e.g., tip projection or vestibular webbing). This element potentially underestimates severity in complex cases. The DRS focuses on nasal indicators because they capture the most persistent and impactful components of UCL/N deformity. Nasal asymmetry, driven by septal deviation and alar displacement, is the primary determinant of perceived cleft severity and directly influences lip aesthetics through aberrant muscle insertions [18–20, 28]. It is proved that nasal restoration is a prerequisite for achieving balanced lip outcomes, as unresolved nasal deformities inherently compromise lip symmetry [18–20, 28]. The scale prioritizes nasal anatomy due to its profound impact on perceived cleft severity and surgical complexity. As a foundation of developing an objective tool, current focus ensures simplicity and reproducibility across diverse settings. The DRS does not directly assess functional outcomes (e.g., airflow), but its anatomical metrics are indicated correlates of functional improvement [18–20, 29, 30]. Future studies may expand to include functional measures.

Although this scale focuses on nasal features, we acknowledge that lip aesthetics play an essential role in overall cleft repair outcomes. Future developments of this system aim to integrate lip-specific parameters to provide a more comprehensive severity and outcome classification framework.

### Interdependence of Nasal and Lip Aesthetics in Cleft Repair

In UCL/N deformity, the nasalis muscle exhibits abnormal insertions, failing to attach to the anterior nasal spine and instead inserting into the nasalis-procerus aponeurosis. This aberrant anatomy disrupts the nasal muscular ring, leading to dislocation of the medial alar cartilages, distortion of the lower lateral cartilage, and significant nasal asymmetry [18–20]. Delaire first described the nasalis muscle's insertion into the anterior nasal spine and septum, a finding later supported by Joos and Friedburg, who demonstrated that unopposed pull by the nasalis muscle causes septal deviation to the non-cleft side [29, 30]. Later, Zide published that the nasalis muscle divides into transverse and alar parts [31]. The transverse nasalis, which joins with the procerus muscle to form the nasalis-procerus aponeurosis, and the alar nasalis, which inserts into the alar cartilage, are essential for stabilizing the nasal framework and influencing lip alignment [25–28].

Talmant and Zheng et al. emphasized the functional importance and complex anatomy of the nasalis muscle, detailing its fixed insertions and four distinct parts, which underscore its critical role in cleft repair [33, 34]. Delaire advocated a wide subperiosteal dissection of facial nasolabial muscles to correct abnormal direction and position of aberrant muscles attachment [32]. Reconstructing the nasalis muscular ring during cleft lip nasal surgery has been shown to reduce alar flaring, narrow the nostril, elevate the nasal sill, and improve contralateral septal deviation, directly influencing both nasal and lip aesthetics [35]. Given its role as the strongest anterior anchorage point of the facial envelope on the cleft side, as described by Veau [36], addressing nasal muscle abnormalities is indispensable for achieving balanced facial aesthetics. This interdependence between nasal and lip structures explains why nasal repair is a prerequisite for optimal lip outcomes.

Fisher’s anatomic subunit cleft lip repair technique not only restores lip height equilibrium but also reconstructs the nasal sill and floor, structures anatomically contiguous with the lip at the nasolabial junction, which are deficient in UCL/N[17]. Subsequent modifications incorporate lateral nasal wall reconstruction to stabilize the alar base and refine the nasal framework [37]. This approach highlights the interdependence of nasal and lip aesthetics: Nasal sill and floor reconstruction during lip repair directly enhances nasal symmetry, while stabilized nasal anatomy ensures balanced lip contours. By addressing these overlapping regions, the technique exemplifies how cleft repair transcends arbitrary anatomical boundaries to achieve holistic facial harmony.

Building on this foundation, the nose, as a central and three-dimensional structure, poses significant reconstruction challenges. Studies have consistently shown it is the most dissatisfying feature of cleft treatment for patients and parents [38–41], with qualitative research confirming poorer aesthetic scores for the nose compared to the lip [42]. The cleft nasal deformity, marked by asymmetry, tissue deficiency, and bone abnormalities, makes cleft lip nasal repair one of the most complex surgeries.

The decision to focus our scoring system on nasal deformity rather than lip deformity is grounded in two key reasons. First, without proper correction of abnormal nasal muscle attachments, lip repair outcomes are often suboptimal. The nasal musculature plays a critical role in stabilizing the lip and maintaining its aesthetic contour. Unresolved nasal deformities inherently compromise lip symmetry and aesthetic results [18–20, 28]. Secondly, multiple studies have identified the nose as the most prominent source of dissatisfaction in cleft repair, consistently yielding poorer aesthetic outcomes than the lip [38–42]. Given its central and three-dimensional nature, nasal deformity significantly impacts overall facial harmony, making it an unavoidable starting point for assessment.

While lip measurements remain important, the nasal scoring system provides a foundational tool to address the most challenging aspects of cleft repair, with future refinements planned to incorporate lip-related parameters for a more comprehensive evaluation.

### Comparison with Existing Scoring Systems

Several existing tools assess cleft nasal deformities but face limitations. Subjective scales like the Anastassov and Chipkov system [11] and Asher–McDade index [43] rely on visual ratings of nasal symmetry or lip alignment, introducing variability between assessors [3, 6, 44–47]. The Pennsylvania Lip and Nose (PLAN) score [42] tracks surgical outcomes but lacks objective anatomical criteria. Kim et al. [48] proposed a quantitative method using angular and spatial nostril measurements, but its complexity requires specialized software, limiting clinical use. Unlike Yao et al.’s cleft width ratio [13], which focuses on a single parameter, the DRS uses three simple, objective measurements, nostril width ratio, columellar angle, alar facial symmetry ratio to eliminate subjective bias. It evaluates critical nasal subunits more comprehensively, aligning with surgical goals to restore balanced anatomy.

### Our Proposed UCL/N Deformity Rating System

Our scoring system employs quantitative measurements to objectively evaluate UCL/N deformity severity, focusing on three critical nasal variables: alar facial asymmetry, nostril width ratio, and columellar angle. These variables categorize primary anatomical severity and predict surgical outcomes and justify surgical outcome. Beyond clinical assessment, the system may serve as a foundation for refining surgical techniques and guiding revision decisions, enhancing both preoperative planning and postoperative evaluation.

The Expected UCL/N Postoperative Outcome Rating Grade is classified as: poor (3), moderate (4-6), good (7-8), excellent (9) (Table 3). We propose a system to justify postoperative outcomes based on the UCL/N DRS, which uses preoperative severity rating score as outlined in (Table 5): Preoperative scale 3-5 is justified to get surgical outcome rated as moderate to poor. Scale 6 should be justified to end up with surgical outcome rated as moderate. Scales 7-9 should be expected to have good to excellent outcome. On the other hand, preoperative scales 7-9 are not justified to get moderate outcome and no acceptance for poor outcome. We also propose the preoperative UCL/N anatomical deformity severity based on the total DRS be graded into Grades I to IV (Table 2).

The "Expected UCL/N Postoperative Outcome Rating Grade" is derived from the preoperative severity score using the proposed rating scale. For cases with a preoperative score of 3 (Grade IV), the expected outcome is graded as "poor," indicating a realistic and conservative expectation given the severe primary deformity. It does not imply no improvement but acknowledges the inherent limitations of surgical correction for cases with severe deformities. We would like to highlight that our grading aligns with the primary deformity severity scale, avoiding overestimation. We intentionally set realistic expectations, refraining from overly optimistic predictions for severely deformed cases. Meanwhile, cases of mild severity are not justified to receive moderate to poor outcomes.

The proposed justification system is not designed to evaluate whether clinical improvement has occurred numerically but rather to determine whether the postoperative outcome is acceptable and aligns with the expected result based on the preoperative severity of the deformity. Its primary purpose is to assess whether the surgical result is "justified" given the severity of the initial condition, without overestimating or underestimating the anticipated outcome. This approach focuses on the appropriateness of the outcome relative to the initial condition, rather than the degree of numerical change achieved through surgery. By identifying cases where the outcome is "not acceptable" or "not justified," the system serves as an objective reference in guiding surgeons to make decision such as the need to refine surgical techniques, address areas for improvement, and optimize patient care. Additionally, it can be used to evaluate the outcomes of individual surgeons, serving as a valuable tool for monitoring and enhancing surgical performance. Deviations from expected outcomes highlight opportunities for technique refinement or training improvement.

All patients in paired analysis underwent primary cheiloplasty using the modified Millard rotation advancement technique. While minor technical variations between surgeons are inevitable, the UCL/N DRS is designed to objectively quantify preoperative anatomical severity and justify postoperative outcomes, irrespective of surgical nuances. This focuses on anatomical fidelity, rather than technique standardization, which demonstrates its utility in real-world clinical practice, where variability exists. While this system could theoretically evaluate outcomes of specific techniques, its robustness for such applications hinges on developing rigorous inclusion and exclusion criteria.

Looking ahead, the UCL/N DRS holds broader potential:

1. *Technique Comparison* By applying the scale to patients treated with different surgical methods, it could objectively compare outcomes across techniques, guiding evidence-based protocol selection.
2. *Competency Benchmarking* When the same technique can be used to evaluate outcomes across surgeons, it is establishing competency benchmarks for training programs and quality assurance.
3. *Surgical Training* As an objective metric, the scale could track trainee progress, ensuring alignment with standardized outcomes and fostering data-driven skill development.

### Broader Applicability and Challenges

While the DRS was demonstrated here for severity classification and outcome justification, future studies could leverage its standardized grading to investigate correlations between deformity tiers and surgical efficacy. For example, large-scale analyses could identify which techniques consistently achieve justified outcomes for specific primary deformities grades. Such data could inform evidence-based surgical algorithms and refine procedural guidelines. As an interesting topic, further assessment may include the potential impact on the surgical technique that the surgeons performed, judging surgical outcome quantitatively, putting aside the surgeon’s personal experiences.

Nevertheless, this objective method may be used to assess and monitor the postoperative aesthetic outcomes of individual surgeon, with any discrepancy from expected outcomes serving as an indicator of potential treatment issues. This system could also be used to assess surgeon’s skills in performing cleft lip surgery and provide the possibility for program monitoring and healthcare system evaluation for cleft care. Program monitoring utilizes the justification system to monitor whether surgical outcomes align with expected severity-based results, ensuring consistency in cleft care. Health care system evaluation may apply it to assess institutional and surgeon’s performance, optimize resource allocation, and refine treatment protocols, driving data-driven improvements in surgical outcomes. Further field testing with a larger cohort will be required to ensure the reproducibility of the system across multiple centers.

In our region, it is rare to encounter cleft patients of Caucasian or Arabic ethnicity, limiting our ability to evaluate the applicability of this rating scale to these specific ethnic groups. Nevertheless, considering the nature of UCL/N deformity, the scale should theoretically be universal across different races or ethnicities. However, further research is required to validate its application across diverse demographic groups.

## Research Limitations and Future Directions

While photographic ratios mitigated distance-related bias, 2D measurements lack the anatomical precision of 3D assessments. Future studies should validate these findings using direct anthropometry or non-invasive 3D imaging modalities, which could avoid practical challenges in pediatric populations while ensuring accuracy.

COVID-19 Movement Control Orders (MCO) restricted paired data collection to 12 pre- and postoperative cases. Though, this paired samples adequately served the purpose of demonstrating the clinical utility of the proposed justification system as a pilot demonstration. While the paired sample size (n=12) limits the generalizability of the outcome justification analysis, the overall study includes 50 preoperative and 50 postoperative cases, which provides adequate statistical power for validating the interrater reliability of the UCL/N DRS. This adheres to the methodological recommendations for studies using ICC according to Bujang et. al [9]. Ongoing studies aim to further validate the outcome justification component in a larger paired cohort

Additionally, despite standardized measurement definitions, subtle interpretive variations may persist due to the shared Malaysian training background of all assessors, particularly residents trained at the same institution. Future studies involving assessors from diverse geographic and institutional training programs are necessary to confirm consistency across clinical settings.

Finally, the classification and justification scoring system currently relies on labor-intensive manual measurements, posing a barrier to clinical adoption. To address this, an AI-driven mobile application is in development to automate analyses, reduce manual effort, and enhance precision.

## Conclusions

The UCL/N DRS proposed in this study is potentially used as a valid and reliable objective tool for evaluating primary UCL/N anatomical deformity, justifying surgical outcome postoperatively. This method of assessment only requires basic instruments and understanding of the anatomical landmarks for operation. It is simple and can be used even in centers with low resources. Good inter-rater agreement validates this scoring system can be used by both plastic surgery residents and expert surgeons. Future developments, including AI integration and expanded field testing, will be needed to further refine and validate the scale, ensuring its utility in both clinical and research settings.

## Appendix 1

**Table 15:** Pre- and postoperative means of nasal deformity variables measured by the assessor groups

Variables	Preoperative mean (SD)		p	Postoperative mean (SD)		p
	Surgeon group	Resident group		Surgeon group	Resident group	
Vertical gap of alar facial grooves (mm)	2.96(1.42)	3.24(1.65)	0.362	1.20(0.88)	1.49(0.96)	0.121
Nasal length (mm)	50.44(5.75)	50.23(5.46)	0.850	56.77(7.61)	56.61(7.49)	0.916
Right nostril width (mm)	14.35(7.13)	14.83(4.81)	0.694	13.17(2.41)	14.06(2.76)	0.091
Left nostril width (mm)	20.88(9.14)	21.21(7.72)	0.847	14.90(3.39)	15.49(3.65)	0.401
Columellar angle (degree)	34.91(10.78)	34.61(13.76)	0.905	10.99(6.69)	13.55(7.12)	0.067

## Appendix 2

**Table 16:** Comprehensive preoperative anatomical severity anaylsis of UCL/N: The table presents the type of cleft for each sample population, the mean assessments for each indicator by six assessors, and the sum of the preoperative rating scale scores based on these mean values, culminating in the final preoperative grade for each sample population

Samples	Type Of Unilateral Cleft Lip	Alar Facial Symmetry Ratio (x̄)	Alar Facial Symmetry Score (x̄)	Nostril Width Ratio (x̄)	Nostril Width Ratio Score (x̄)	Collumelar Angle (x̄)	Collumelar Angle Score (x̄)	Σ Preoperative Nasal Rating Sevevery Score (x̄)	UCL/N Deformity Severity Grade (x̄)
1	CCLP	0.08	2	0.34	1	40.83	1	4	III
2	CCLP	0.07	2	0.35	1	43.50	1	4	III
3	CCLP	0.098	2	0.41	1	34.25	1	4	III
4	CCLP	0.03	3	0.42	1	32.33	1	5	III
5	ICL	0.04	3	0.62	2	25.83	2	7	II
6	CCLP	0.09	2	0.32	1	42.17	1	4	III
7	CCLP	0.103	1	3.71	1	63.33	1	3	IV
8	CCLP	0.07	2	2.50	1	45.67	1	4	III
9	CCLP	0.06	2	0.47	1	38.33	1	4	III
10	CCLP	0.04	3	0.45	1	41.75	1	5	III
11	CCLP	0.102	1	3.90	1	56.67	1	3	IV
12	CCLP	0.08	2	2.67	1	37.67	1	4	III
13	CCLP	0.04	3	0.44	1	43.75	1	5	III
14	CCLP	0.06	2	0.32	1	31.92	1	4	III
15	ICL	0.04	3	1.13	2	34.25	1	6	III
16	CCLP	0.03	3	0.48	1	45.83	1	5	III
17	CCLP	0.09	2	0.39	1	31.17	1	4	III
18	ICL	0.03	3	1.18	2	20.92	2	7	II
19	CCLP	0.09	2	0.45	1	41.25	1	4	III
20	CCLP	0.15	1	0.36	1	33.67	1	3	IV
21	ICL	0.095	2	0.50	1	36.67	1	4	III
22	ICL	0.03	3	1.11	2	12.42	3	8	II
23	ICL	0.06	2	1.43	1	24.42	2	5	III
24	CCLP	0.07	2	0.35	1	52.42	1	4	III
25	ICLA	0.06	2	2.18	1	35.67	1	4	III
26	CCLP	0.04	3	0.45	1	31.33	1	5	III
27	ICL	0.04	3	0.62	2	20.83	2	7	II
28	ICLA	0.07	2	1.21	2	19.50	2	6	III
29	CCLP	0.07	2	3.90	1	34.67	1	4	III
30	ICLA	0.04	3	0.56	1	25.75	2	6	III
31	ICL	0.01	3	0.61	2	13.92	3	8	II
32	CCLP	0.07	2	1.85	1	51.83	1	4	IV
33	ICLA	0.04	3	1.73	1	24.83	2	6	IV
34	ICL	0.03	3	0.69	2	16.25	2	7	II
35	CCLP	0.09	2	0.42	1	48.50	1	4	III
36	ICLA	0.06	2	0.68	2	29.67	2	6	III
37	CCLP	0.05	2	0.53	1	28.08	2	5	III
38	CCLP	0.07	2	0.41	1	38.83	1	4	III
39	CCLP	0.05	3	0.41	1	32.83	1	5	III
40	CCLP	0.07	2	0.44	1	40.83	1	4	III
41	CCLP	0.03	3	0.56	1	28.67	2	6	III
42	CCLP	0.14	1	0.34	1	48.58	1	3	IV
43	ICLA	0.06	2	1.44	1	18.58	2	5	III
44	ICLA	0.08	2	1.45	1	26.33	2	5	III
45	ICLA	0.03	3	0.83	2	11.92	3	8	II
46	CCLP	0.025	3	0.56	1	49.50	1	5	III
47	CCLP	0.042	3	0.49	1	47.92	1	5	III
48	ICLA	0.08	2	1.99	1	31.92	1	4	III
49	ICL	0.06	2	1.57	1	23.67	2	5	III
50	CCLP	0.07	2	0.44	1	46.67	1	4	III

*CCLP* Complete cleft lip and palate; *ICL* Incomplete cleft lip; *ICLA* Incomplete cleft lip with alveolus
